# Preliminary Study on the Connection Between the Mineral Profile of Horse Hooves and Tensile Strength Based on Body Weight, Sex, Age, Sampling Location, and Riding Disciplines

**DOI:** 10.3389/fvets.2021.763935

**Published:** 2022-03-07

**Authors:** Gabriel Rueda-Carrillo, René Rosiles-Martínez, Anaid Ireri Hernández-García, Einar Vargas-Bello-Pérez, Francisco J. Trigo-Tavera

**Affiliations:** ^1^Universidad Nacional Autónoma de México, Facultad de Medicina Veterinaria y Zootecnia, Departamento de Nutrición Animal y Bioquímica, Ciudad Universitaria, Mexico City, Mexico; ^2^Department of Veterinary and Animal Sciences, Faculty of Health and Medical Sciences, University of Copenhagen, Copenhagen, Denmark

**Keywords:** equine hoof, minerals, tensile strength, hoof quality, riding discipline

## Abstract

Hoof mineral profile is important as it could affect locomotion. Factors such as body weight, sex, age, and riding disciplines affect hoof mineral profile. In Mexico and globally, studies are needed on the characterization of mineral profile of hooves and tensile strength, as this could help to prevent overgrowth or microfractures. Therefore, in the present survey, 165 samples of equine hoof cuttings from different sex, ages, breeds, and riding disciplines from different regions of Mexico were analyzed for their mineral composition, and a universal testing machine was used to measure tensile strength. More than half of the samples were from males (63%) and aged 3 to 5 years (52%). Most samples were obtained from horses used for reproduction (36%) and working (29%) purposes. The most preponderant minerals were K (3,416 μg/g), Na (2,242 μg/g), and Ca (631 μg/g). Tensile strength ranged from 1.2 to 45 N. Females had higher (*P* < 0.05) amounts of Zn than males. Animals younger than 3 years old have lower (*P* < 0.05) levels of Na than those between 3 and 5 years old. Horses used for reproduction had lower (*P* < 0.05) Mg concentrations than animals used for running and working. Tensile strength was similar between demographic characteristics. Horses from Santa Gertrudis military ranch had higher (*P* < 0.05) levels of Ca, Se, and Na compared with horses from other sampling location. Copper was higher (*P* < 0.05) in horses from racecourse. Potassium was higher (*P* < 0.05) in horses from the Presidential General Staff. Overall, there was no evident connection between sex, ages, breeds, and riding disciplines from different regions of Mexico and the mineral composition of the hoof or its tensile strength. Further research should focus on the relation on specific feeding regimes, horse individual characteristic, hoof mineral contents, and tensile strength.

## Introduction

Members of the *Equidae* family represent the result of a digitigrade evolution, as they rest on a finger, protected by a horn tissue covering called a hoof (1), which is divided into three parts: the wall, the sole, and the frog. The hoof wall is a highly keratinized structure that does not have blood vessels or nerves, as it is generated from the continuous cell division of a germ layer of basal cells, and provides protection for internal structures covering from the coronary border to the ground (2). The structure of the sole is similar to that of the horny layer of the wall, since it is composed of vertical keratin tubules; however, the sole is softer than the wall since it has a higher degree of humidity, and approximately one-third part of this structure is made up of water (3). Its main function is to protect the internal sensitive structures; however, the outer perimeter of the sole provides support, distributing part of the horse's weight with the hoof wall (4). The frog, also formed by keratinized tissue and a higher percentage of water, has greater flexibility than that of the rest of the hoof, and its main function is to carry out the hemodynamic flow.

The chemical characteristics of the horse's hoof depend on the body's nutrition, and its reflection will be part of the quality of the feed supplied (5). In this regard, an adequate supply of nutrients to the keratin-forming cells is essential for the quality and function of the hoof tissue. If nutrition is compromised, due to deficiency or excess of nutrients, it can cause delay or overgrowth of the hoof (6); other hoof problems associated with this can be thin walls and soles, microfractures (quarters), wall peeling, and interrupted growth patterns on the hoof wall (7). The growth rate and hoof chemical composition are of vital importance, as this affects ability to perform locomotion (8). Depending on the horse's genetics, diet, exercise, and the surrounding environment, the average growth rate is 0.6 to 1.3 cm/month (9).

Minerals comprise approximately 4% of body weight and are an essential part of the horse's diet as they help to metabolize proteins, fats, and carbohydrates; aid muscles and nerves to function properly; maintain the acid–base balance of body fluids; and are essential components of each enzyme required for metabolic functions (5). Horses require at least 15 different mineral elements, and some of these are required in relatively greater amounts such as calcium (Ca), phosphorus (P), sodium (Na), potassium (K), chlorine (Cl), magnesium (Mg), and sulfur (S), while microminerals such as iron (Fe), copper (Cu), zinc (Zn), manganese (Mn), and selenium (Se) are needed for keratinization in the living part of the hoof (10); however, with regard to microminerals, Zn and Cu are also important for improving hoof quality (11). That is why equine hoof characteristics such as its tensile strength, wear, and integrity, are associated with the chemical composition of the hoof and clearly depends on dietary nutrients (10–12).

Biomechanically, the purpose of the hoof is to transfer a large proportion of the ground reaction force between the hoof wall and the skeleton, which means that the hoof modulates irregularities in externally applied loads and attenuates the impact of its own contact with the hoof sole (13). Tensile testing measures the resistance of a material to a slowly applied quasi-static loading. A sample of any material is placed in the universal testing machine where tension and compression tests can be carried out. The effort obtained in the highest applied force is the tensile strength, which determines the force needed to break a sample. This value is also commonly known as the maximum tensile strength or breaking strength (14). In Mexico, data on the mineral profile of horse hooves and tensile strength is not available. However, this information is of vital importance for horse owners as it could be used to prevent health problems. Therefore, the objective of this survey was to determine the connection between sex, ages, breeds, and riding disciplines from different regions of Mexico and the mineral composition (Ca, K, Fe, Mg, Se, Zn, Na, and Cu) of the hoof or its tensile strength.

## Materials and Methods

### Animals

**Figure 1:**
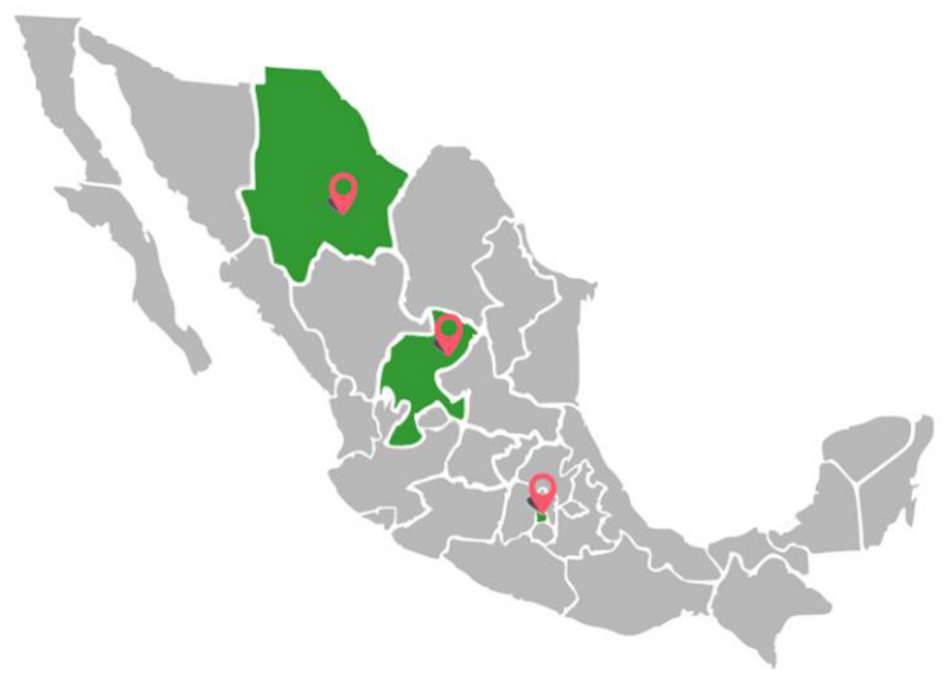
A map of Mexico showing the three sampling locations.

Hoof trim samples were obtained from 165 horses (37% female and 63% male) without apparent locomotion problems and clinically healthy, aged 4.8 ± 3.1 years, with a mean body weight of 441 ± 68 kg. Samples were taken from three locations: (1) Mexico City (CDMX) [private ranch, mounted police, Presidential General Staff (PGS), racecourse, and military school, *n* = 82], (2) Chihuahua (Santa Gertrudis military ranch, *n* = 33), and (3) Zacatecas (private ranch, *n* = 50) (Figure 1).

Age was divided in categories using 33% and 66% percentiles (low values <33%, mean values 33–66%, and high values >66%); thus, three categories were obtained: <3 years, 3–5 years, and >5 years. Most of the animals chosen for the present study were young animals because it is the age range used for surveillance or working, and the surveyed horses belong to this riding discipline. Demographic data from surveyed horses is shown in Table 1.

**Table 1:** Demographic data from surveyed horses.

**Characteristic**		**Frequency**	**Percentage**
		**(*n*)**	**(%)**
Sex	Male	104	63
	Female	61	37
Age	<3 years	47	28.5
	3 to 5 years	52	31.5
	>5 years	34	20.6
Breed	Crossbreed (unknown breeds)	50	30.3
	Pure blood	34	20.6
	Spanish	14	8.5
	Warmblood	28	17.0
	Quarter horse	27	16.4
	Azteca	11	6.7
Sampling	Private ranch (Zacatecas)	50	30.3
location	Santa Gertrudis military ranch	33	20.0
	Mounted police	22	13.3
	Presidential general staff	18	10.9
	Private ranch (Mexico City)	14	8.50
	Military school	18	10.9
	Racecourse	10	6.10
Riding	Reproduction	59	35.8
discipline	Working	47	28.5
	Surveillance	26	15.8
	Racing	10	6.10
	Dancing show	14	8.50
	Maintenance	6	3.60
	Dressage	3	1.80

### Hoof Samples

Equine hoof samples (65 ± 21 g) were obtained from hoof trimmings routinely performed on horses during shoeing and/or trimming. Each sample was obtained from the right forelimb, from the area of the wall, from the bearing surface. Before trimming, each hoof was cleaned with a wire brush, then scrubbed with deionized water. Upon collection, clippings from each hoof were placed into labeled plastic bags and stored for subsequent analyses (15).

**Figure 2:**
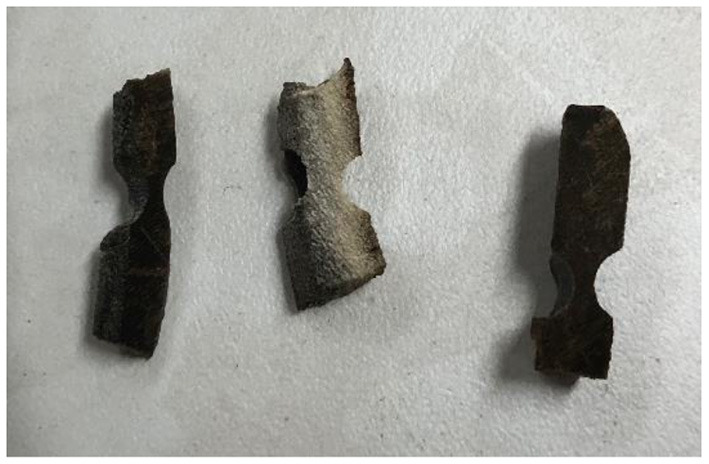
Notched hoof samples.

For tensile strength determination, samples were sliced to a uniform thickness of 0.5 ± 0.1 mm and later cut to 5 × 0.05 cm. Then, samples were notched at their central area using a Dremel tool (Dremel^®^ Illinois, USA) to reach 2 mm of thickness. Samples that were not broken during notching were not considered for analysis because they did not have enough thickness for analysis. The rest of each hoof wall clipping was repackaged into labeled plastic bags and stored at room temperature until processed for mineral analysis (15) (Figure 2).

### Mechanical Testing

**Figure 3:**
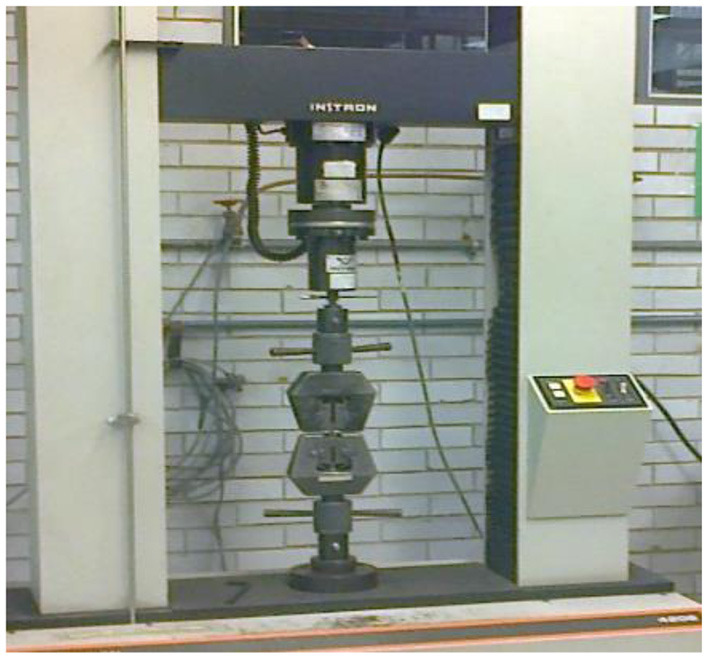
Instron universal testing machine.

The hoof tensile strength was measured with an Instron universal testing machine (Model 4206, Massachusetts, USA) with a load capacity of 5 kilonewtons (KN) (Figure 3) and an acceleration of 2 mm/min. This instrument allowed to measure hoof tensile strength, and results are expressed as N, which is the number of kilograms over square millimeter required to break the sample. This measurement was achieved when a millimeter portion of the hoof trim (between 10 and 40 mm^2^) is held at the ends and subjected to tension until it breaks (15). All analyses were carried out during the first 48 h after collection, to ensure that the samples did not dehydrate. Samples had a dry matter content of 99%, and all samples had the same handling and the same collection-testing time.

### Mineral Profile

In order to determine the association, the hoof mineral elements (Ca, K, Fe, Mg, Se, Zn, Na, and Cu) were measured from hoof trimming samples. Firstly, organic matter was removed, and then samples were pulverized and digested with nitric acid at 70°C for 12 h (15) and then subjected to atomic absorption spectrometry (Perkin Elmer 3100 Atomic Absorption Spectrometer, Waltham, Massachusetts, US). Samples from these studies were from animals that were monthly trimmed; therefore, samples from some animals were not enough to be used for mineral analysis.

### Statistical Analysis

Depending on the type of variables, descriptive statistics (mean, standard deviation, frequencies, percentages, and 95% confidence intervals for the means) were obtained for the mineral elements, tensile strength, and demographic characteristics of surveyed horses. To test for normality, the Shapiro–Wilk test was performed. A fixed-effects generalized linear model (GzLM) (16) was used to compare the different characteristics of the horses. In the model, the dependent variables were minerals and tensile strength, and the remaining variables (sex, breed, age, sampling location, and riding discipline) were considered as factors. The differences (*P* < 0.05) among means were compared with Bonferroni test.

Principal component analysis (PCA) with the Varimax criterion and canonical discriminant analysis (CDA) were performed for mineral elements and tensile strength (17–19). Due to lack of information, some variables or categories of variables did not enter the analyses. Statistical analyses were performed with IBM SPSS Statistics® Version 27.

## Results

Potassium and Na were the most abundant minerals in hoof samples, while Cu was observed in lower quantities (Table 2). The tensile strength was 17.09 ± 10.14 N/mm^2^ (CI: 14.56–19.63 N/mm^2^) (Table 3). Due to the amount of available hoof sample, it was not possible to analyze the total amount of some minerals.

**Table 2:** Main mineral contents (μg/g) from hoof samples.

**Mineral**	***n***	**Mean ±standard deviation**	**95% confidence interval**
Potassium	115	3,416 ± 3,270	2,812–4,020
Sodium	115	2,242 ± 1,102	2,038–2,446
Calcium	165	631 ± 418	566–695
Magnesium	132	181 ± 98.1	164–198
Iron	119	114 ± 145	88.5–141
Zinc	165	79.1 ± 55.0	70.6–87.6
Selenium	83	29.9 ± 34.3	22.4–37.4
Copper	37	1.80 ± 1.04	1.46–2.15

**Table 3:** Comparison of mineral elements with equine characteristics.

**Variable**	***n***	**Mineral elements (μg/g)**								**Tensile strength (N/mm^2^)**
		**Ca**	**Mg**	**Se**	**Fe**	**Zn**	**K**	**Na**	**Cu**	
**Sex**										
Male	104	679 ± 437	189 ± 94	34 ± 35	125 ± 163	73 ± 51^b^	2,968 ± 3,049	2,135 ± 1,067	1.76 ± 0.89	16 ± 10
Female	61	550 ± 374	166 ± 105	26 ± 34	102 ± 119	89 ± 60^a^	4,530 ± 3,576	2,508 ± 1,161	1.91 ± 1.39	22 ± 10
**Age**										
<3 years	47	602 ± 385	172 ± 109	20 ± 23	124 ± 168	89 ± 57	3,927 ± 3,077	1,875 ± 1,006^a^	1.91 ± 0.94	20 ± 10
3–5 years	52	525 ± 413	151 ± 107	33 ± 41	113 ± 134	78 ± 59	5,228 ± 4,154	3,038 ± 1,042^b^	2.93 ± 1.51	17 ± 0.4
>5 years	34	831 ± 563	235 ± 78	44 ± 27	113 ± 134	73 ± 52	3,706 ± 3,032	2,267 ± 1,086^ab^	1.49 ± 1.19	22 ± 10
**Breed**										
Azteca	11	999 ± 238	281 ± 69		82 ± 114	111 ± 25	2,101 ± 554	1,301 ± 304	1.50 ± 1.12	20 ± 14
Pure blood	34	828 ± 389	206 ± 40	55 ± 29	147 ± 187	64 ± 56	3,826 ± 3,168	2,364 ± 1,016	2.03 ± 1.12	21 ± 6
Warmblood	28	805 ± 569	203 ± 58	68 ± 35	135 ± 145	21 ± 27	6,412 ± 3,958	3,253 ± 962		
Spanish	14	672 ± 141	163 ± 89		50 ± 21	79 ± 72	867 ± 301	978 ± 545	1.90 ± 0.46	15 ± 13
Crossbred	50	492 ± 290	169 ± 97	8 ± 3	109 ± 145	98 ± 39	1,674 ± 680	2,096 ± 701	2.02 ± 1.03	15 ± 7
Quarter horse	27	250 ± 108	145 ± 126	8 ± 3	104 ± 131	108 ± 52				
**Riding discipline**										
Dressage	3	916 ± 152	231 ± 7^ab^		45 ± 39	115 ± 18	1,769 ± 182	1,296 ± 155	2.07 ± 0.79	14 ± 9
Growing	6	915 ± 169	313 ± 65^ab^		45 ± 28	124 ± 15	2,122 ± 544	1,122 ± 243	1.61 ± 1.10	20 ± 14
Racing	10	788 ± 294	204 ± 43^a^		147 ± 225	127 ± 36	1,532 ± 502	1,206 ± 184	2.22 ± 0.99	20 ± 5
Surveillance	26	721 ± 240	227 ± 64^ab^		132 ± 132	83 ± 36	1,646 ± 695	2,137 ± 683	2.16 ± 1.08	16 ± 9
Dance	14	672 ± 141	163 ± 89^a^		50 ± 21	79 ± 72	869 ± 301	978 ± 545	1.90 ± 0.46	15 ± 13
Reproduction	59	593 ± 532	131 ± 89^b^	30 ± 36	114 ± 134	69 ± 55	5,271 ± 3,758	2,759 ± 921	0.38 ± 0.41	22 ± 10
Working	47	529 ± 412	182 ± 118^a^	29 ± 33	128 ± 162	71 ± 59	564 ± 3,663	3,216 ± 945		
**Sampling region**										
Mexico City	82	740 ± 255^b^	215 ± 70		103 ± 146	90 ± 44^a^	1,558 ± 713^a^	1,771 ± 771^a^	2 ± 1	17 ± 10
Chihuahua	33	947 ± 592^a^		63 ± 34^a^	143 ± 152	6 ± 6^b^	8,034 ± 2,447^b^	3,414 ± 917^b^		
Zacatecas	50	244 ± 97^b^	126 ± 112	8 ± 3^b^	105 ± 141	111 ± 46^ab^				

*^a−c^Different literals indicate significant differences (P < 0.05)*.

Table 3 shows mineral contents and tensile strength from hoof samples with regard to the different demographic characteristics of surveyed horses. Females had higher (*P* < 0.05) amounts of Zn than males. Animals younger than 3 years old have lower (*P* < 0.05) levels of Na than those between 3 and 5 years old. Horses used for reproduction had lower (*P* < 0.05) Mg concentrations than animals used for running and working. Horses from Chihuahua had higher (*P* < 0.05) levels of Ca, Se, K, and Na compared to horses from other sampling locations, but lower Zn. Due to amounts of sample, Cu was only analyzed from CDMX, and the values were higher for racecourse (2.22 ± 0.99 μg/g) and the lowest for the mounted police (1.59 ± 1.29 μg/g) (*P* < 0.05). Tensile strength (*P* > 0.05) was similar between all demographic characteristics. Horses from Santa Gertrudis military ranch had higher (*P* < 0.05) levels of Ca, Se, and Na compared to horses from other sampling locations. Potassium was higher (*P* < 0.05) in horses from the Presidential General Staff.

**Figure 4:**
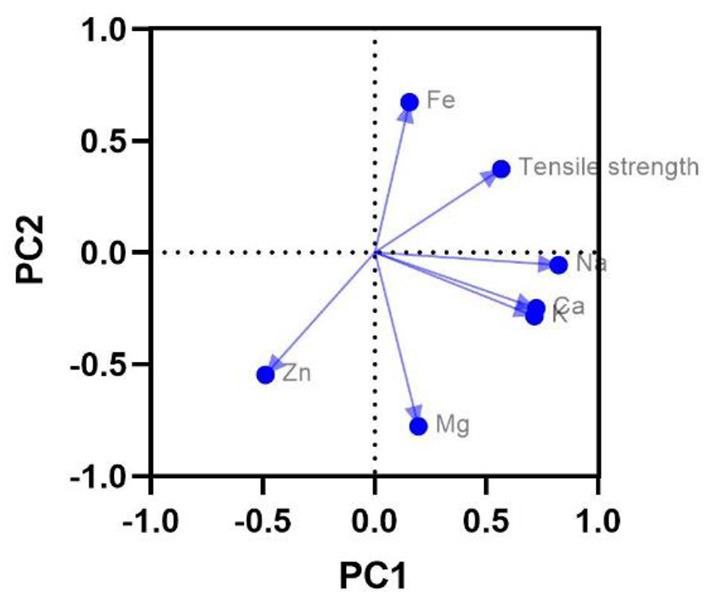
Component plot of mineral elements and tensile strength of horse hooves.

The PCA resulted with two significant components where the Kaiser test explained 56.62% of total variation. Table 4 shows the results of Varimax rotation. The first factor explaining 32.29% of the variability was identified by six of seven variables (Ca, Mg, Zn, Na, and tensile strength), and the second factor (24.33% of the variability) was identified by four of seven variables (Mg, Fe, Zn, and tensile strength) (Figure 4).

**Table 4:** Varimax rotation factor scores for the two-factor model for minerals in horse hooves.

**Mineral elements (μg/g)**	**Components**	
	**1**	**2**
Sodium	0.80	−0.19
Potassium	0.77	0.05
Calcium	0.76	0.02
Magnesium	0.42	0.68
Tensile strength (N/mm^2^)	0.42	−0.53
Iron	−0.06	−0.69
Zinc	−0.30	0.67
Variability (%)	32.29	24.33

**Figure 5:**
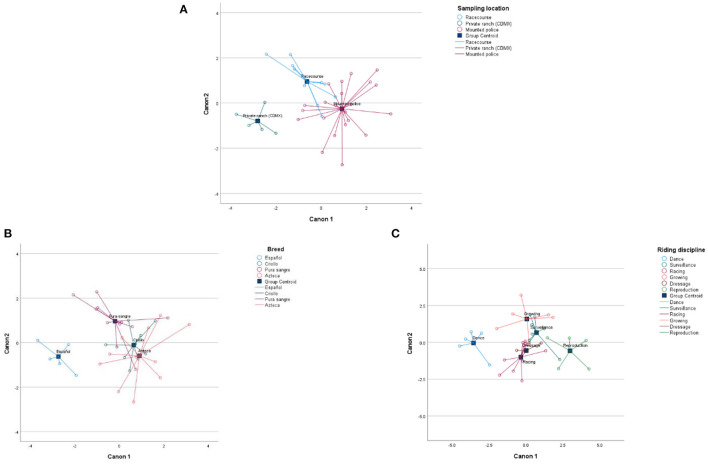
Bi-plots of different mineral elements according to sampling location **(A)**, breed **(B)**, and riding disciplines **(C)**.

The raw canonical coefficient of canonical discriminant functions by sex, age, breed, sampling location, and riding discipline was significant (*P* < 0.05) (Table 5). In canonical 1 (sampling location), 81.90% of the total variation was explained, and the most discriminating minerals were Ca, K, and Na with a positive correlation and Zn with a negative correlation. In canonical 2, 18.10% of the total variation was explained, and the most discriminating variables were Fe and tensile strength with a positive correlation and Mg with a negative correlation. Figure 5A shows that the private ranch (CDMX) was isolated from the rest of the sites by the lower influence of minerals in canonical 1, but more discriminated by Zn.

**Table 5:** Raw canonical coefficient of canonical discriminant functions.

**Item**	**Sampling location (*n* = 37)**		**Breed (*n* = 36)**			**Riding discipline (*n* = 37)**				
	**C1**	**C2**	**C1**	**C2**	**C3**	**C1**	**C2**	**C3**	**C4**	**C5**
Ca	**0.31**	**–**0.24	0.34	**−0.39**	**–**0.21	0.24	0.14	**–**0.26	0.46	**−0.57**
Mg	0.14	**−0.83**	0.08	**−0.77**	0.27	**–**0.21	**0.77**	**–**0.27	0.46	0.02
Fe	0.03	**0.34**	0.07	0.27	**0.30**	0.08	**–**0.17	0.40	**0.52**	0.12
Zn	**−0.31**	**–**0.13	**–**0.34	0.006	**0.49**	**–**0.24	0.02	0.02	0.32	**0.49**
K	**0.72**	**–**0.24	**0.64**	**–**0.17	0.56	**0.62**	0.38	**–**0.32	0.12	0.56
Na	**0.38**	0.16	0.41	0.08	**−0.44**	0.44	**–**0.25	**–**0.28	0.40	**−0.52**
Tensile strength	0.46	**0.49**	**0.50**	0.40	**–**0.20	0.35	0.11	**0.86**	**–**0.11	**–**0.29
Eigenvalue	1.82	0.40	1.58	0.50	0.03	3.64	1.00	0.26	0.05	0.01
Variance total (%)	81.90	18.10	74.7	23.8	1.50	73.30	20.20	5.20	1.00	0.30
Wilk's lambda	0.25			0.25				0.80		
*P*-value	<0.001			0.006				<0.001		

*Bold values denote elements belonging to each canonical element*.

For the breed trait, canonical 1 explained 74.7% of the total variation, and the most discriminating elements were as follows: K and tension strength having a positive correlation, and in canonical 2, the most discriminating elements were Ca and Mg having a negative correlation, and 23.80% of the total variation was explained. Figure 5B shows that the Spanish breed was found to be isolated from the rest of the breeds that entered the analysis, due to the lower influence of minerals found in canonical 1.

For the riding disciplines, canonical 1 described 73.30% of the total variation, with K as the most discriminating mineral with a positive correlation. Canonical 2 explained 20.20% of the total variation, with Mg as the most discriminating mineral with a positive correlation. Canonical 3 explained 5.20% of the total variation, with Fe as the most discriminating mineral with a positive correlation. Canonical 4 described 1%, being Fe has a positive correlation and is the most discriminating mineral, while canonical 5 explained 0.30% of the total variation, having the following as the most discriminating minerals: Zn with a positive correlation and Na and Ca with a negative correlation. Figure 5C shows that horses used for dancing show and reproduction were isolated from the rest of the riding disciplines, due to the lower influence of minerals found in canonical 1.

## Discussion

The hoof structure is very important for horse welfare and performance, and therefore, results obtained in this study seek to expand the knowledge and existing values on the mineral contents of horses' hooves, in addition to tensile strength values (20–22). Until today, studies on this topic from horses in Latin America are scarce. It is important to note that the animals from which the hoof sample was taken for this study had a macroscopic “healthy” external appearance, but no histological analysis was performed. Further studies should determine histology analysis to confirm hoof healthiness.

With regard to the concentration of minerals determined by age, horses under 3 years of age had higher levels of Zn compared to horses over 3 years of age, but Na levels were lower in younger horses. Zinc is a key mineral for keratinization process. The keratin cells of the upper part of the hoof lose their nucleus and organs; during this process, the cells die and form the corneal structure of the hoof (23). Zinc content in this study was higher in females than in males. In a study carried out in Iran (10), no relationship was not observed between Zn content and hoof quality. However, Tocci et al. (20) compared hoof quality of Anglo-Arab horses and Monterufoli ponies and reported that Anglo-Arab horses that had stronger hooves tended to accumulate more Zn; however, in a study with donkeys' hooves (24), a negative relationship between hoof strength and Zn content was reported. Some researchers have chosen to study the effect of adding Zn to horse's diet. Most of these studies showed that Zn supplementation increases hoof quality for up to 1 year (10). Hepworth et al. assured that animals supplied with Zn and biotin could have improved hoof structure (25). In this regard, the hoof condition seems to be closely related to dietary nutrient (5).

Another factor that could affect hoof structure is the working hours that horses are subjected to, which implies physical and, therefore, physiological wear and tear. Not meeting nutritional requirements can lead to bone or tissue pathologies of hooves (26, 27). In the study, animals used for reproduction had lower Mg concentrations compared to those used for racing and work. Ozana da Silva and Manso Filho (28) measured the concentration of some blood minerals from mares in maintenance, pregnancy, lactation, and at parturition. Lower values of Mg among pregnant and maintenance mares were reported, which could explain that in the hoof, rearing mares have lower values compared to race and working horses. Those differences could be due to the physiological wear and tear that pregnancy represents (28). Thus, further studies should consider blood hormone profiling together with diet analysis.

In the present study, sampling location was a factor that affected some mineral content in horse hooves. For example, Zn was higher in the Santa Gertrudis military ranch compared to the rest of the sampling locations, while Na was lower from samples obtained from a private ranch located in Mexico City. According to FAO, in arid and semi-arid areas such as the Santa Gertrudis military ranch located in Chihuahua State, there is usually an accumulation of exchangeable Na in the soil, which clearly increases Na content of the pastures in those regions (29). In this study, when using CDA, a separation of the samples from the private ranch from Mexico City was also observed with regard to the rest of the sampling locations. The effect of the sampling location has been reported in other species, for example, a study with donkey's hoof showed Fe levels of 437 ± 65 ppm, and that was attributed to the grazing conditions that the animals had, in addition to high rainfall around the days of sampling. The authors mentioned that this could promote a highly acidic environment with consequent Fe solubilization, causing a greater absorption of this mineral into the hoof (24).

Although in the present study no significant differences were found using a generalized linear model between breeds, when using CDA, it was observed that the canonical variables Ca, Mg, Fe, Zn, K, Na, and the tensile strength were separated between breeds, mainly associated with Spanish breed. Other studies have shown that hoof mineral contents vary between breeds and between species. For example, sole contents of Ca were higher in Anglo-Arab horses (1,088 ± 133 ppm) compared with Monterufoli Pony (373 ± 144 ppm) (20). In another study, higher hoof Zn values (114 ± 2 μg/g) from ponies fed with a diet based on pellets and gelatin were reported (27), than those measured on donkey cuttings where Na values obtained were 267 ± 15 ppm (24).

It is important to consider that when carrying out this type of studies, the specific hoof area for sampling can yield to differences in mineral levels. For example, Mg levels obtained in this study were 181 ± 98 μg/g, while de Souza et al. (30) reported different Mg values from the wall (960 ± 631 ppm) and the frog (1,389 ± 768 ppm). Similarly, Tocci et al. (21) reported Mg values for the hoof wall of 373 ± 33 ppm and 255 ± 38 ppm for the sole. These discrepancies can be attributed to the fact that mineral contents will vary depending on the hoof sampling area, and therefore, during sampling, it is important to use the same sites in order to be comparable in the same study.

In this study, K and Na were the most preponderant minerals in hoof samples. In this study, Na was 2,242 ± 1,102 μg/g, while other studies (31) have reported lower values (250 ± 135 ppm). The K levels determined (3,416 ± 3,270 μg/g) were higher than those mentioned by Sargentini et al. (1,690 ± 112 ppm) (24) and de Souza et al. (460 ± 425 ppm) (31). With regard to Mg, values of 181 ± 98 μg/g were obtained, while in mules and donkeys, de Souza et al. (11) indicated values of 840 ± 922 ppm and 1,178 ± 855 ppm, respectively.

Calcium is a mineral to which more attention must be paid, as it helps to create cross-links of sulfur between the proteins of the hoof, allowing cohesion between cells (27). The stronger the cohesion, the healthier and more impenetrable the hoof will be. In the present study, there were values of 631.27 ± 418 μg/g, and de Souza et al. obtained values in the wall area of 178 ± 137 ppm, while in the frog, they were 366 ± 180 ppm (31). This is an indicative that even in the same structure, there are micro-zones with different concentrations of mineral elements.

In the case of Fe, a value of 114 ± 145 μg/g was obtained, which is below the values (290.58 ± 256.26 ppm) reported by de Souza et al. (30). With regard to Cu, an average of 1.80 ± 1.04 μg/g was obtained. This value was somewhat similar to that obtained by de Souza et al., 3.51 ± 1.6 ppm (30), but it was found below (4.3–4.53 ppm) that described by Faria et al. (32). In another study, by de Souza et al., Cu values of 5.61 ± 1.38 ppm were recorded (11). Copper has a very specific role in the growth of the hoof, as it is essential to build the keratin bridges present in the hoof. The sulfur in methionine and cysteine serves as a structural component that forms these bridges, and a copper-dependent enzyme in cells is responsible for building junctions between various proteins. If Cu is deficient, enzyme activity decreases, and the structural bridge is compromised. Ultimately, it is likely that the growth of these protein bridges will be affected, and in turn, this causes the hoof to show cracks from the growth (11).

On the other hand, the metabolic inter-relationship (antagonistic or synergistic relationship) that exists between mineral elements must be considered (33). For example, in this study, zinc had an antagonistic relationship with selenium. From the PCA results, it was observed that in PC1, Ca, Ma, Zn, Na, and the tensile strength had a negative correlation with zinc. On the other hand, in PC2, there was a positive correlation together with Mg, Fe, and tensile strength. These relations should be further studied and also relate them to animal's feed.

Bertram and Gosline (7) stand out for being pioneers in the study of hoof tensile strength and concluded that the properties of keratinous materials are strongly influenced by their state of hydration. This could explain the difference between Geyer values (40 to 60 N/mm^2^) (26) and those obtained in the present study ranging from 1.2 to 45 N/mm^2^, probably because of different environmental, seasonal, and geographical conditions from the sampling locations. In a study in which tensile strength of 20 mares between 4 and 20 years old was measured, values of 21.70 to 35.32 N/mm^2^ were reported (15). Some of those mares were pregnant, and probably that could be a factor that affects tensile strength, and thus, this should be considered when interpreting results on tensile strength as well as animal's age.

When performing PCA and CDA, the tensile strength was introduced as a variable, obtaining positive correlations in both tests. This suggests that there is a relationship between these variables and mineral elements. In this regard, Zenker et al. (34) reported values of hoof tensile strength between 60.3 and 68.3 N/mm^2^ and coronary edge tensile strength from 52.6 to 62.9 N/mm^2^. This indicates that the hoof has areas that deform differently with the horse's gait; therefore, it is important to try to have these structures in the best possible state, since poor conformation can cause growth abnormalities and development, which predisposes to hoof microfractures. Several studies (35–37) have shown that microfractures are the second most recurrent hoof problem. On the other hand, it has to be considered that in those studies, the measurement of tensile strength was performed *post mortem* using tendons, ligaments, and cartilage (38–40), while in the present study, samples were obtained from hooves of live animals.

Lastly, carrying out these types of studies is difficult due to the sample amounts that can be obtained per animal. It must be considered that the animals have different conditions such as feeding regime, handling, and geographical location, and that is why it is necessary to have a well-defined inclusion criterion, which will lead to a greater number of samples and more robust results for mineral contents in horse hooves.

## Conclusions and Perspectives

Results from this survey offer a first profile and established a reference for mineral profile from Mexican horses with different characteristics. Overall, there was no evident connection between sex, ages, breeds, and riding disciplines from different regions of Mexico and the mineral composition of the hoof or its tensile strength. Further research should focus on the relation on specific feeding regimes, horse individual characteristic, hoof mineral contents, and tensile strength.

## Data Availability Statement

The raw data supporting the conclusions of this article will be made available by the authors, without undue reservation.

## Ethics Statement

Ethical review and approval was not required for the animal study because samples were collected from private owners and no animal manipulation was involved. Written informed consent for participation was not obtained from the owners because samples obtained were when horse hooves were trimmed, owners just allowed us to remove the small pieces from trimmed hooves.

## Author Contributions

RR-M contributed to conception and design of the study. GR-C organized the database and made the sampling and processing of the studied materials. AH-G performed the statistical analysis. GR-C and EV-B-P wrote the first draft of the manuscript and sections of the manuscript. FT-T is the main tutor of the study and he reviewed the results obtained and the manuscripts. All authors contributed to the article and approved the submitted version.

## Funding

This study was funded by the Universidad Nacional Autónoma de México, Facultad de Medicina Veterinaria y Zootecnia, Departamento de Nutrición Animal y Bioquímica.

## Conflict of Interest

The authors declare that the research was conducted in the absence of any commercial or financial relationships that could be construed as a potential conflict of interest.

## Publisher's Note

All claims expressed in this article are solely those of the authors and do not necessarily represent those of their affiliated organizations, or those of the publisher, the editors and the reviewers. Any product that may be evaluated in this article, or claim that may be made by its manufacturer, is not guaranteed or endorsed by the publisher.
